# Deep Brain Stimulation of Nucleus Accumbens Region in Alcoholism Affects Reward Processing

**DOI:** 10.1371/journal.pone.0036572

**Published:** 2012-05-22

**Authors:** Marcus Heldmann, Georg Berding, Jürgen Voges, Bernhard Bogerts, Imke Galazky, Ulf Müller, Gunther Baillot, Hans-Jochen Heinze, Thomas F. Münte

**Affiliations:** 1 Department of Neurology, University of Magdeburg, Magdeburg, Germany; 2 Department of Neurology, University of Lübeck, Lübeck, Germany; 3 Department of Nuclear Medicine, Medical School Hannover, Hannover, Germany; 4 Department of Stereotactic Neurosurgery, University of Magdeburg, Magdeburg, Germany; 5 Department of Psychiatry, University of Magdeburg, Magdeburg, Germany; The University of Melbourne, Australia

## Abstract

The influence of bilateral deep brain stimulation (DBS) of the nucleus nucleus (NAcc) on the processing of reward in a gambling paradigm was investigated using H_2_[^15^O]-PET (positron emission tomography) in a 38-year-old man treated for severe alcohol addiction. Behavioral data analysis revealed a less risky, more careful choice behavior under active DBS compared to DBS switched off. PET showed win- and loss-related activations in the paracingulate cortex, temporal poles, precuneus and hippocampus under active DBS, brain areas that have been implicated in action monitoring and behavioral control. Except for the temporal pole these activations were not seen when DBS was deactivated. These findings suggest that DBS of the NAcc may act partially by improving behavioral control.

## Introduction

Positive and negative reinforcement are assumed to be key mechanisms in the acquisition and maintenance of drug addiction [1]. While negative reinforcement drives motivated behavior during withdrawal, positive reinforcement takes place during the early stages of addiction, where alcohol/drug intake leads to pronounced release of dopamine and assigns, via reward driven learning mechanisms, “incentive salience” to drug associated cues. In turn, these cues can then elicit a strong and uncontrollable desire for a drug [2], resulting in cue-induced craving, one major reason for the high relapse rates in addiction treatment. The nucleus accumbens (NAcc) and the extended amygdala, comprising the central nucleus of the amygdala (CEA), parts of the NAcc's medial shell, and the bed nucleus of the stria terminalis (BSTM), are known to be crucial for these addiction-related reinforcing mechanisms. Here we report a patient, whose alcohol addiction was treated successfully with DBS affecting the NAcc, BSTM and the ventral pallidum (VP). Besides its involvement in the moderation of stress induced responses to acute withdrawal the BSTM plays a major role in the modulation of reinforcement related dopaminergic activity via its excitatory connections to the VTA [3] and is seen as one element within an extended brain reward circuitry [4]. The NAcc has been described as a limbic-motor interface, that integrates contextual information from the hippocampus, emotional information from the amygdala and information of goal-directed behavior from the prefrontal cortex [5]. There is increasing evidence from functional and clinical investigations for Goto and Grace's [6] limbic-motor interface model. By recording local field potentials in humans recent studies reported the NAcc's involvement in action control [7], processing of reward [8], and unexpected stimuli [9]. Based on the assumption that a dysfunctional motivation and reward system is one pathogenic factor for several psychiatric disorders like OCD, depression or addiction, the NAcc became a target area for treating the above mentioned diseases successfully with DBS [10], [11], [12] or ablation of the NAcc [13], [14]. The transmission of input from the NAcc to brainstem motor-related targets is only one aspect of the VP's functional relevance within the processing of reward. It is also known as a convergence point for input from reward related sites like prefrontal cortex, amygdala and VTA [15]. Furthermore, VP neurons are involved in the motivational transformation of predictive information provided by conditioned stimuli into incentive salience [16]. To study how the DBS treatment impacts the processing of rewards in the brain, we examined this patient while he engaged in a gambling paradigm using H_2_[^15^O]-PET (positron emission tomography). Given the central position of the stimulation site within the reward processing matrix, we expected changes in blood flow in parts of this network but also in distant cortical areas.

## Methods

### Ethics Statement

DBS treatment was conducted as part of an off-label study protocol approved by the ethical review board of the University of Magdeburg. PET scanning was performed with approval of the ethical review board of the Medical School Hannover. The patient gave written informed consent before the beginning of the first scanning session. Consent to publication was obtained from the patient as well.

### Patient

The patient, a 38 year old man, had started to drink alcohol at age 11. By the age of 18 he fulfilled the DSM-IV criteria for alcohol dependence. His first detoxification treatment was at age 15. Multiple detoxification and prolonged withdrawal therapies as well as anti-craving therapy with acamprosate had been unsuccessful. Before surgery the longest period of abstinence lasted 3 months. During these drug-free intervals the patient reported massive craving and high sensitivity to alcohol-related cues. Pre- and post-surgical assessment included Symptom Check list 90 (SCL), psychopathology, obsessive-compulsive drinking scale (OCDS), alcohol urge questionnaire (AUQ). The alcohol dependence scale (ADS) was only assessed before surgery. In addition, the patient had also been examined with a comprehensive neuropsychological test battery, which had revealed neither marked neuropsychological difficulties nor dementia. One week after implantation of the DBS electrodes (13 January, 2008) the stimulation was switched on. The patient experienced a short period of hypomania, which stopped upon changing stimulation parameters. Since then up to the submission of this report the patient has been alcohol abstinent and reports a virtually complete reduction of his sensitivity to alcohol related cues.

Bilateral stereotactically guided implantation of quadripolar brain electrodes (model 3387, Medtronic, Minneapolis, MI, USA) was performed in general anesthesia as described by Heinze et al. [17]. The electrode position was confirmed intraoperatively using stereotactic X-rays and finally by computed tomographic imaging (CT, see figure S1). Postoperative CT-scans were retransferred into treatment planning MRI images. The most distal contact of the electrode was located 1–2 mm rostral to the anterior commissure projecting onto the lateral border of the NAcc. This particular placement was necessary to save a prominent A1-segment of the anterior cerebral artery running through the intended target area. Settings of the impulse generator (Kinetra®, Medtronic, Minneapolis) at time of testing were: monopolar cathodic using the most distal contact in each hemisphere (frequency: 130 Hz, pulse width: 90 µs, amplitude: 3.5V [17]). The current (radius of approx. 3 mm around the active electrode contact [18]) affected dorsal parts of the NAcc, the BSTM and the VP (see figure S1).

### Gambling paradigm

In order to investigate the impact of DBS on reward-processing and risk-taking we used an adapted version of a gambling task [19]. In each trial of the task two numbers, 5 and 25, were presented (see figure S2). By pressing a mouse button with the right index or middle finger the corresponding number was selected. After the response, one of the numbers turned into red, the other into green. If the selected number became green (red), the corresponding amount in Eurocent was won (lost). If the response-time exceeded 1 second, both numbers turned into gray. On some trials gains or losses were doubled. The patient was instructed to win as much money as possible. The patient got feedback regarding his current balance after each run and was paid off at the end of the session. Each session comprised 6 runs containing mostly losses (L) during the active scanning period and 6 runs with mostly wins (W). The order was WLLWWLLWWLLW for the first session (DBS on) and LWWLLWWLLWWL for the second (DBS off). All runs started with 12 trials with a 50∶50 chance to win. In the subsequent 56 trials the winning chance was either 75∶25 (W) or 25∶75 (L). Each trial lasted 2.5 seconds.

### PET scanning and procedure

The PET scanning was carried out 18 month after DBS implantation. Two sessions comprising 12 runs/tracer-injections each were performed. After the first session during which the stimulator was active, the generator was switched off and 90 min later the second session was started. At the end of the second session DBS was reactivated. The patient was blind to the generator status.

An ECAT EXACT 922/47 PET-Scanner (Siemens, Erlangen, Germany) with a total axial field of view of 162 mm and a spatial resolution of 7–8 mm (full width at half maximum) in reconstructed tomograms was used for data acquisition. At the beginning of the session a transmission scan of 10 min was performed using Ge-68 rod sources. Thereafter the regional distribution of cerebral radioactivity was recorded always after bolus injection of 740 MBq O-15 water (H_2_[^15^O]) per run). Each injection started after the first 12 trials of a run, i.e. when the winning chance turned to either 75∶25 or 25∶75. The 3D-acquisition of a 90s PET-frame started 15 seconds after tracer injection.

### PET analysis

**Figure 1:**
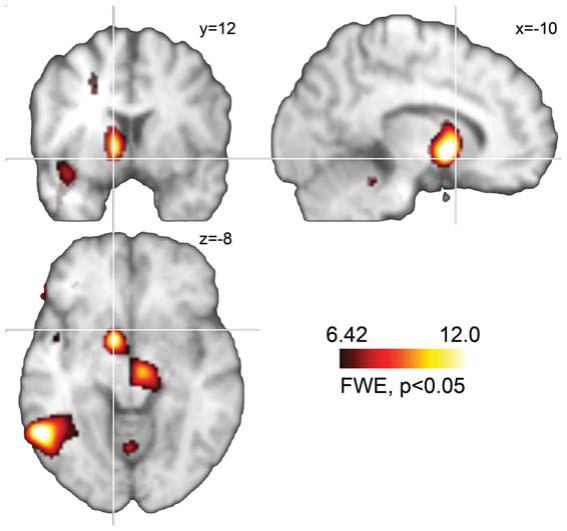
Contrast image for the comparison DBS on>DBS off. Crosshair position indicates the location of the nucleus accumbens according to MNI standard coordinates. Activations are corrected for multiple comparisons (FWE = 0.05; cluster threshold 50 voxel). See table 1 for the corresponding list of activations.

After iterative reconstruction statistical calculations and image processing was performed with Matlab 7.2 (The Mathworks Inc., Natick, MA). For realignment, image normalization and statistical mapping we used the PET-module of SPM2 [20]. Interscan head movement was corrected by realigning all PET scans of one session to the session's first scan. Afterwards, the two resulting mean relative rCBF images were normalized to standard MNI space. The parameters stemming from the normalization procedure were then used to normalize all PET images of each session. Thereafter, images were smoothed by applying a 16 mm Gaussian low-pass filter. The resulting voxel size in standardized stereotactic space was 2×2×2 mm^3^. For statistical analysis the single subject analysis of the PET module was used, with one factor comprising the four levels “DBS on win condition”, “DBS on loss condition”, “DBS off win condition” and “DBS off loss condition”. The reported effects were obtained by calculating linear contrasts. All reported activations exceeded a threshold of p<0.005 (uncorrected) and a cluster size >50 voxels. The global contrast DBS on>DBS off (figure 1 and table 1) was computed with a stricter statistical threshold (FWE-correction, p<0.05, cluster size >50 voxels).

**Table 1:** Significant activations resulting from the comparison DBS on > DBS off (FWE 0.05, cluster-threshold 50 voxel).

on>off	hemis-phere	Z-values	MNI coordinates
medial globus pallidus	left	6.88	−10 6 −6
thalamus, ventral posterior medial nucleus	right	6.78	16 −18 −2
frontal lobe (white matter)	right	6.42	22 0 28
middle temporal gyrus	left	6.80	−56 −54 −8
superior temporal gyrus	left	6.50	−44 −26 6
temporal lobe (white matter)	left	6.49	−36 −48 16
occipital lobe	left	6.76	−40 −86 40
inferior frontal gyrus	left	6.51	−38 18 −20
middle frontal gyrus	left	5.84	−46 26 −30
inferior frontal gyrus	left	5.51	−56 34 −6
Cerebellum	right	5.82	6 −60 −20
lateral occipital cortex	right	5.73	60 −62 42
inferior frontal gyrus	right	5.48	38 32 −20
parietal lobe, postcentral gyrus	right	5.45	62 −20 24
lateral occipital cortex, cuneus	right	5.39	26 −74 36
Cerebellum	left	5.39	−22 −82 −26
occipital fusiform gyrus	left	5.09	−22 −86 −16
middle occipital gyrus	left	5.33	−40 −82 12
inferior occipital gyrus	left	4.77	−42 −88 2
Cerebellum	left	5.27	−36 −48 −48
inferior frontal gyrus	left	5.14	−50 24 22
middle frontal gyrus	left	5.13	−30 2 52
frontal lobe (white matter)	left	4.94	−24 8 40

## Results

### Behavior

**Figure 2:**
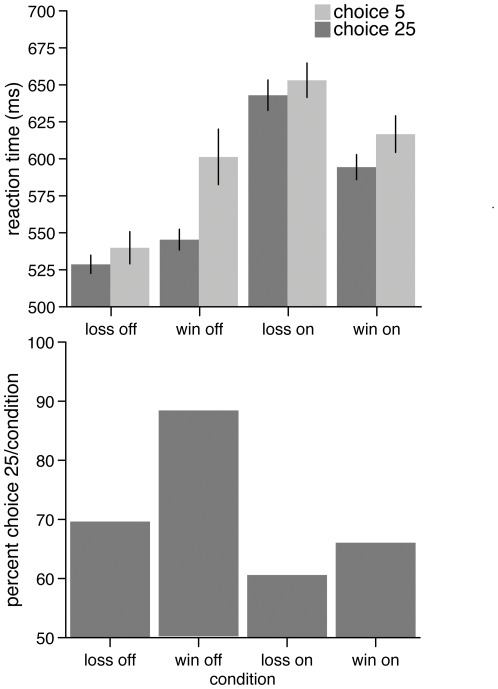
Behavioral data. Upper panel: Reaction times for the “5” and “25”-selections for each condition. Lower panel: percent choices for the “25”-selection for each condition.

DBS status had a marked effect on choice behavior and response speed. Since in the present paradigm selecting 25 instead of 5 results in an overall 50%/50% chance in winning/losing 25 Eurocent, choosing 25 is considered as the riskier choice [23]. Active DBS was associated with somewhat slower and less risky choices, implying a more impulsive, riskier and less controlled behavior when neural activity was not modulated by DBS (figure 2). To test for order effects, the patient was examined again four months later using the identical paradigm with reversed order of DBS status (1^st^ session off, no PET scan). Although the differences were much smaller compared to the previous scanning session, the tendency to make more risky choices in the off compared to the on condition still remained (win/off 58.6%, win/on 54.3%, loss/off 57.4%, loss/on 59.1%). Reaction times were in the on-condition generally faster than in the off-condition and thus showed an inverse pattern of results compared to the first investigation (see figure S3 for additional results).

### DBS global effect

Contrasting active against inactive DBS conditions resulted in prominent activations in the left medial globus pallidus, the left temporal and frontal lobe as well as in the right ventral posterior medial nucleus of the thalamus (see figure 1, for a detailed list of activations see table 1). Noteworthy, this comparison revealed no rCBF changes in the NAcc, BNST or ventral pallidum, i.e. those regions targeted by the applied DBS.

### DBS paradigm related rCBF changes

**Figure 3:**
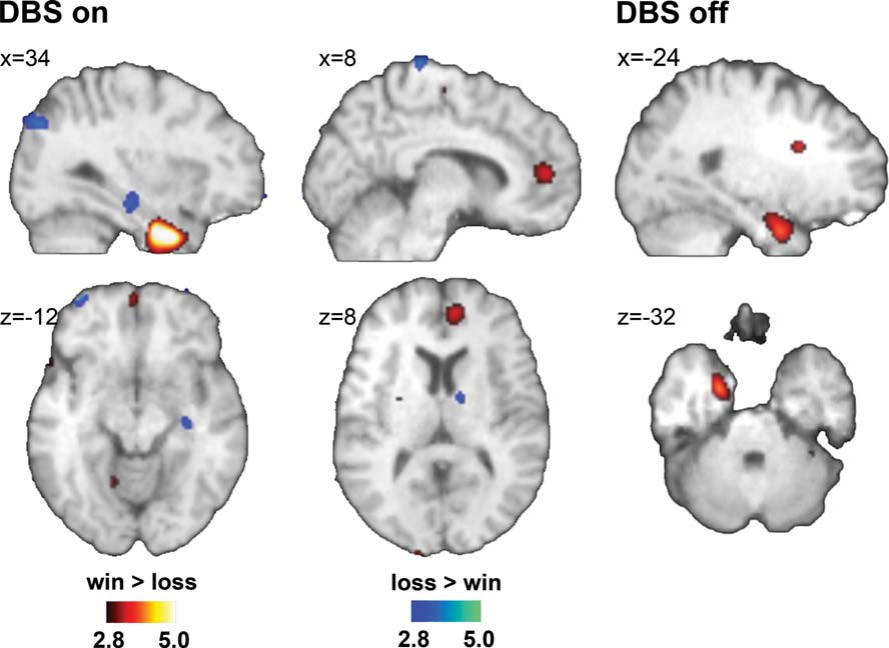
Comparisons win>loss and loss>win with active and inactive DBS. Contrast images for the comparisons win>loss (color scale red/yellow) and loss>win (color scale blue/green) with active (left panel) and inactive (right panel) DBS in the target area. First level statistical analysis was performed with p<0.005 (uncorrected) and 50 voxel cluster threshold. Except for activation at the temporal pole no activation shown for the DBS on condition remained statistically significant when DBS was off.

With active DBS the win condition (relative to losses) caused pronounced activations in the paracingulate cortex (BA32) and the temporal poles bilaterally, whereas losses showed significantly more activity in the precentral gyrus, the frontal pole, the hippocampus and the precuneus (see figure 3 and table 2).

**Table 2:** Significant activations related to the comparisons win vs. loss condition with DBS on (win on>loss on, loss on>win on) and win vs. loss condition with DBS off (win off>loss off, loss off>win off).

	hemis-phere	Z-values	MNI coordinates
**win on > loss on**			
temporal fusiform cortex, anterior division	right	4.45	34 0 −36
temporal pole, superior temporal gyrus	left	4.40	−50 14 −32
fusiform cortex	left	4.14	−26 0 −46
inferior frontal gyrus	left	3.40	−54 22 −2
paracingulate cortex	right	4.10	8 50 8
superior frontal gyrus	right	3.00	2 34 56
lingual gyrus	left	3.00	−16 −58 −8
frontal pole	right	2.96	0 64 −18
**loss on > win on**			
middle frontal gyrus	left	4.10	−42 28 50
precentral gyrus	left	3.60	−18 −20 78
precentral gyrus	right	3.60	12 −28 78
frontal pole	left	3.46	−36 62 −10
superior frontal gyrus	left	3.21	−26 48 −26
frontal pole	left	2.94	−34 58 −18
parietal lobe	left	3.26	−20 −40 42
superior occipital gyrus	right	3.18	−34 80 34
parahippocampal gyrus	right	3.00	32 −20 −16
**win off > loss off**			
parahippocampal gyrus, temporal pole	left	3.39	−22 2 −34
hippocampal gyrus	left	3.14	−28 −6 −24
nucleus caudatus	right	3.29	18 6 10
white matter	left	3.05	−24 14 22
occipital pole	left	2.89	−12 −98 4
**loss off > win off**			
middle frontal gyrus	left	3.75	−42 24 26
posterior cingulated gyrus	right	3.57	2 −28 26
superior frontal gyrus	right	3.43	8 26 62
frontal pole	right	3.36	2 64 −8
frontal pole	right	2.78	24 70 −4
superior temporal gyrus	left	3.27	−62 −4 6
frontal lobe (white matter)	right	3.13	24 8 30
parietal lobe (white matter)	right	3.13	16 −46 14
inferior parietal lobe	right	3.04	54 −38 54
postcentral gyrus	right	2.85	40 −30 72
middle occipital gyrus	left	2.83	−52 −78 2
middle occipital gyrus	left	2.64	−58 −76 10

With stimulator turned off, the win-associated activation in the paracingulate cortex disappeared and that of the temporal poles decreased remarkably. Likewise, the loss-related activations of the hippocampus and the precuneus were no longer seen.

## Discussion

This case study provides evidence, that DBS affecting the NAcc/BSTM/VP region has an impact on reward processing. Behaviorally, the patient showed a tendency towards more risky behavior when the stimulator was turned off. A similar behavioral pattern is known from Parkinson patients treated with drugs affecting dopaminergic D2/D3 receptors [21], [22] known to give rise to a number of impulse control disorders [21] but also from studies with healthy young subjects receiving dopaminergic D2/D3 agonists [23], [24], [25]. Thus, one might speculate that DBS in the NAcc normalizes reward processing and reduces impulsive choices in patients with chronic alcohol abuse. However, this interpretation has to be substantiated by further experiments, since a re-examination outside the PET-scanner four month later did not result in a full replication of the behavioral results . While the pattern of risky choices still remained by trend, the reaction times showed an inverse pattern of results. Thus, we are not able to rule out any order effects entirely, although this had been the re-examination's intention. The question remains why the behavioral results could not be fully replicated.

Importantly, robust and statistically significant changes in the PET activation maps were observed that were more pronounced with stimulators turned on. Specifically, monetary rewards (compared to losses) led to an activation of the paracingulate cortex and the temporal poles. The paracingulate cortex integrates affective and motor information in behavioral control and adaptation [26] in particular in economic decisions [27], [28] and receives input from the NAcc [7], [29]. Functionally, the paracingulate cortex can be divided into two sections: the dorsal part, which is known to be involved in the processing of cognitive control, and the rostral part, which is related to the processing of affective information in behavioral control tasks [26], [30], [31]. The increased activity in the rostral part of the paracingulate cortex under active DBS is pointing to an involvement of emotional processes in behavioral adaptation and control in the win condition. The participation of emotional processes is known to be essential for effective adaptation and control of behavior [32], [33], [34], [35]. The absence of rCBF changes in this part of the paracingulate cortex under deactivated DBS suggests that without active DBS this integrative function is not involved. In conjunction with the observed behavioral data this implies that DBS in the NAcc complex seems to improve behavioral adaption. The temporal poles receive input from all three senses [36] and, as part of the paralimbic circuitry and the parahippocampal cortex, are interconnected to the amygdala, the orbitofrontal cortex, and the hippocampus [37], [38]. Accordingly, the temporal poles are often described as a multimodal convergence zone integrating sensory input, memory and emotion in order to bind emotional information across sensory domains [39], [40]. This temporal pole function forms the basis for more complex cognitions like autobiographical memory [41] or the processing of self-referential information [41], [42], [43]. Self-referential information is needed for evaluative judgment [44], and results - successful decisions and adequate self-attribution assumed - in self-conscious emotions like joy or pride [45]. As indicated by the increased rCBF changes, these mechanisms are likely triggered to a greater extent in runs with an excess of win-trials. Again, the differences between win and loss trials are more pronounced for the DBS-on than for the DBS-off condition, indicating that under active DBS positive outcomes of choices increase self-referential processing. Accordingly, DBS effects may facilitate the selective ascription of positive outcome to one's own behavior. By contrast, for the runs with more loss trials greater activation was seen in the precuneus and hippocampus. The latter has been implicated in reward based learning and decision making processes [46]. The processing of reward related contextual aspects in the hippocampus [47] improves and facilitates predictions about upcoming events [48], [49]. As Coricelli and colleagues [50], [51] have shown, hippocampal and parahippocampal areas also support the affective evaluation of the outcome of a decision. The hippocampal activations observed in the loss condition under active DBS suggest an increased involvement of these evaluation processes. The precuneus has been tightly linked to evidence accumulation in decision making situations [52], in particular in unpredictable situations [53]. Accordingly, the precuneus is also reported to be active during risky decisions [54], [55] and the identification of risk [56]. With respect to the present investigation active NAcc-DBS seems to increase activation in cortical areas which are necessary for identifying situations that are (potentially) disadvantageous.

Interestingly, no blood flow changes were observed in the DBS target area. This might be caused by the partial volume effect in PET imaging, which results in an underestimation of the activity in small structures like the NAcc or BSTM [57].

To sum up, under stimulator on conditions, brain areas were seen activated under active DBS that have been previously linked to aspects of behavioral control and decision making. Importantly, under deactivated DBS most of these activations were no longer seen with the exception of the right temporal pole. This said, it has to stressed that the present PET and behavioral data are coming from a single case and thus have to be interpreted with caution. Due to ethical reasons it was not practicable to examine the patient a second time and accordingly potential order effects cannot be ruled out. However, the reported results fit well to the literature and provide a first glimpse at the impact of DBS on the neural underpinnings of decision making and cognitive control. Together with the behavioral effect towards more risky behavior this suggests that behavioral control is impaired with the stimulator turned off. Future investigations have to examine the hypothesis that enhanced behavioral control is likely to contribute to the clinical effect of DBS in the NAcc.

### Conclusion

Despite the known limitations of single case reports we conclude that DBS in the NAcc improves behavioral control in decision making processes by activating areas related to processing of self-referential information, integration of emotional information and updating of contextual information. While further investigations are needed to substantiate this finding, this mechanism might contribute to the efficacy of DBS in addiction.

## Supporting Information

Figure S1 **Positioning of DBS electrodes.** Transversal reconstruction of T1 MRI after image fusion with postoperative stereotactic CT (bottom line) indicating the final position of the active electrode contact (hyperintense CT-signal). The upper line shows the position of the active electrode contact (indicated by X) in projection onto coronal slices (1.3 mm rostral to AC (right electrode) and 2.0 mm rostral to AC (left electrode)) of an atlas of the human brain^1^. Overlayed in light yellow is the current spread. Abbreviations: AC: anterior commissure; BSTM: Bed nucleus of stria terminalis; EGP: external globus pallidus; AcC: nucleus accumbens, central (subventricular) part (core).
(TIF)

Figure S2 **Schematic representation of the paradigm.** In the depicted trial, the participant selected “25” by pressing the left mouse button. As the “25” turned into green, the participant has won 25 Euro-Cent.
(TIF)

Figure S3 **Behavioral data of the re-examination session.** Upper panel: Reaction times for the “5” and “25”-selections for each condition. Lower panel: percent choices for the “25”-selection for each condition.
(TIF)
