# Supplementary figures and images for: Deep Brain Stimulation of Nucleus Accumbens Region in Alcoholism Affects Reward Processing
Source: PLoS One. 2012 May 22;7(5):e36572. doi: 10.1371/journal.pone.0036572 (PMC3358316; doi:10.1371/journal.pone.0036572)

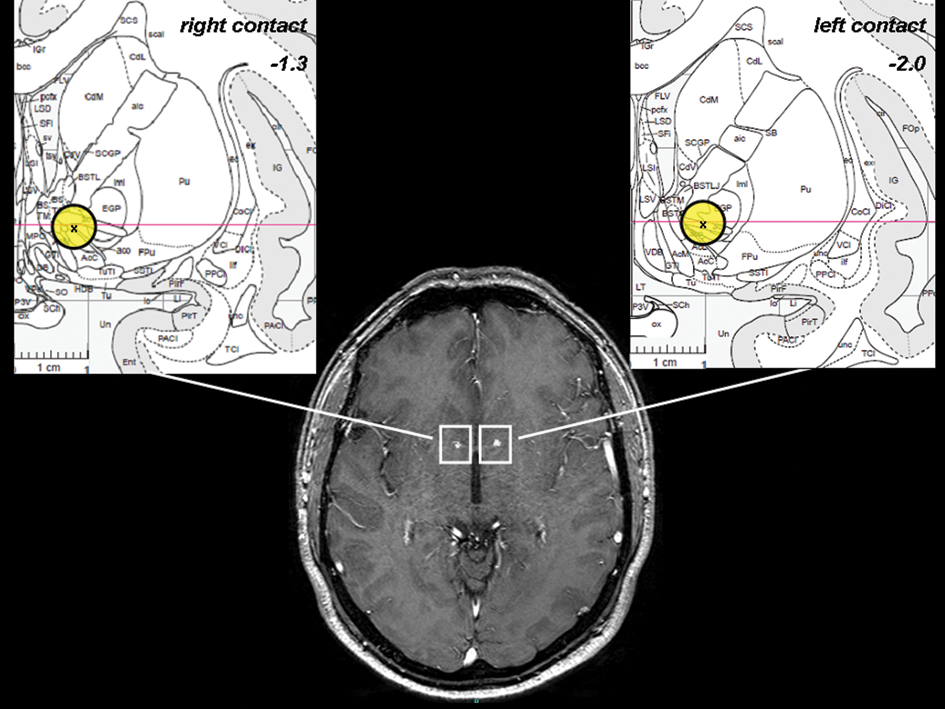

Supplement: Figure S1 — Positioning of DBS electrodes. Transversal reconstruction of T1 MRI after image fusion with postoperative stereotactic CT (bottom line) indicating the final position of the active electrode contact (hyperintense CT-signal). The upper line shows the position of the active electrode contact (indicated by X) in projection onto coronal slices (1.3 mm rostral to AC (right electrode) and 2.0 mm rostral to AC (left electrode)) of an atlas of the human brain1. Overlayed in light yellow is the current spread. Abbreviations: AC: anterior commissure; BSTM: Bed nucleus of stria terminalis; EGP: external globus pallidus; AcC: nucleus accumbens, central (subventricular) part (core). (TIF) [file pone.0036572.s001.tif]

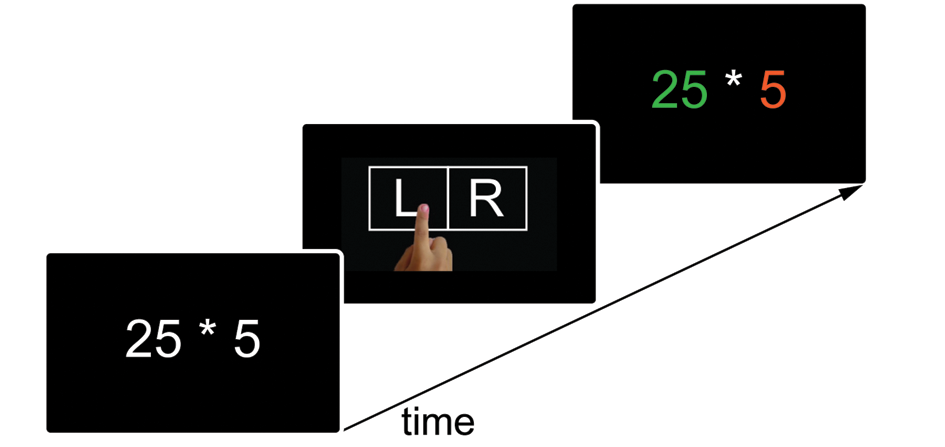

Supplement: Figure S2 — Schematic representation of the paradigm. In the depicted trial, the participant selected “25” by pressing the left mouse button. As the “25” turned into green, the participant has won 25 Euro-Cent. (TIF) [file pone.0036572.s002.tif]

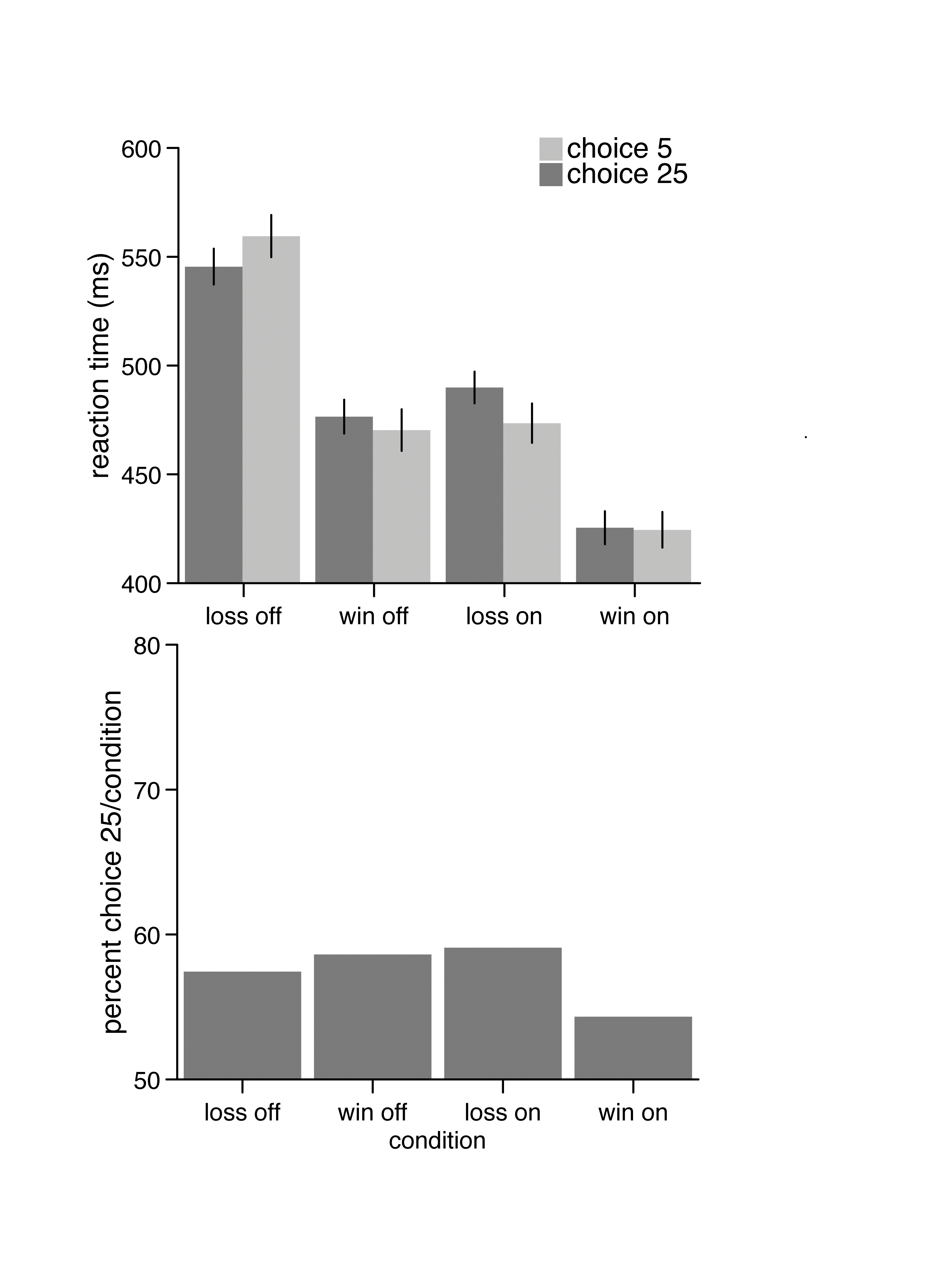

Supplement: Figure S3 — Behavioral data of the re-examination session. Upper panel: Reaction times for the “5” and “25”-selections for each condition. Lower panel: percent choices for the “25”-selection for each condition. (TIF) [file pone.0036572.s003.tif]
